# Experimental Study of Back Wall Dross and Surface Roughness in Fiber Laser Microcutting of 316L Miniature Tubes

**DOI:** 10.3390/mi9010004

**Published:** 2017-12-26

**Authors:** Erika García-López, Alexis G. Medrano-Tellez, Juansethi R. Ibarra-Medina, Hector R. Siller, Ciro A. Rodriguez

**Affiliations:** 1 Tecnológico de Monterrey, Escuela de Ingeniería y Ciencias, Monterrey, Nuevo León 64849, Mexico; garcia.erika@itesm.mx (E.G.-L.); a.medranotellez@gmail.com (A.G.M.-T.); juansethi@itesm.mx (J.R.I.-M.); 2 Department of Engineering Technology, University of North Texas, Denton, TX 76207, USA; hector.siller@unt.edu

**Keywords:** vascular stents, fiber laser, AISI 316L stainless steel, microcutting, back wall dross, surface roughness

## Abstract

Laser cutting is a key technology for the medical devices industry, providing the flexibility, and precision for the processing of sheets, and tubes with high quality features. In this study, extensive experimentation was used to evaluate the effect of fiber laser micro-cutting parameters over average surface roughness ($R_{a}$) and back wall dross ($D_{\mathrm{bw}}$) in AISI 316L stainless steel miniature tubes. A factorial design analysis was carried out to investigate the laser process parameters: pulse frequency, pulse width, peak power, cutting speed, and gas pressure. A real laser beam radius of 32.1 μm was fixed in all experiments. Through the appropriate combination of process parameters (i.e., high level of pulse overlapping factor, and pulse energy below 32 mJ) it was possible to achieve less than 1 μm in surface roughness at the edge of the laser-cut tube, and less than 3.5% dross deposits at the back wall of the miniature tube.

## 1. Introduction

Biomedical applications such as micro shavers, needles, biopsy instruments, and coronary stents require the use of fiber laser for cutting miniature tubes [1,2]. These medical applications are highly demanding in terms of dimensional tolerance and surface topography. Therefore, there is great interest in the proper characterization of this kind of novel micro-manufacturing processes with medical grade materials. In micromanufacturing, laser technology provides high flexibility due to its capability for processing a wide range of materials [3]. However, some of the issues considered in high precision manufacturing include slag, burrs, heat affected zones, and dross adhesion. These product quality problems are reduced with secondary post-processing techniques such as electropolishing and by adjusting cutting process parameters such as pulse frequency, pulse width, peak power, cutting speed, gas pressure and stand-off distance.

The research literature shows a number of studies related to fiber laser cutting. However, documented research regarding quality features of fiber laser cutting at micrometric scale is relatively limited. Karatas et al. [4] presented the influence of laser beam waist position relative to the workpiece surface and workpiece thickness on kerf size and striation pattern in high-strength low-alloy steel (HSLA-steel). Radovanovic et al. [5] examined the effect of peak power and material thickness on surface roughness in 1 mm, 3 mm, and 6 mm thick low-carbon sheets. Sobih et al. [6] presented the impact of cutting speed over surface roughness and striations patterns in the fiber laser cutting of mild steel sheets. Ahn et al. [7] reported the influence of cutting speed on surface roughness in Inconel 718 sheets of 1 mm, 1.6 mm, and 2 mm in thickness using an neodymium-doped yttrium aluminium garnet (Nd: YAG) laser. Baumeister et al. [8] performed fiber laser cuts in 100 µm, 200 µm, and 300 µm steel sheets (AISI 304), using N_2_ and O_2_ assist gases and modifying cutting speed. Preusch et al. [9] used a fiber laser for the 3D machining of alumina substrates, modifying pulse overlap and repetition rate as the most influencing parameters in order to achieve appropriate surface roughness. Kleine et al. [10] established cutting speed, and laser pulse length as the significant model terms which improve surface roughness in the fiber laser cutting of AISI316L stainless steel material after running an analysis of variance (ANOVA). Similarly, Muhammad et al. [11] explained that surface roughness is minimized by reducing peak pulse power, and increasing pulse frequency.

Also, some analytical models have been developed in order to predict laser melting phenomenon and dross adherence. Tani et al. [12] developed an analytical model relating the physical properties of material with melt film geometry for dross adhesion prediction in the laser cutting of steel. Yilbas et al. [13] reported a mathematical model of dross formation according to material properties such as viscosity, density, surface tension, and cutting properties such as gas velocity and liquid layer thickness. Furthermore, some experimental work has been done to understand dross adherence in laser cutting. Adelman et al. [14] concluded that gas pressure and focus position are significant parameters for burr height. Pfeifer et al. [15] reported the high impact of gas flow rate on cutting performance according to nozzle stand-off distance. Kathuria [16] explained that dross is adhered to the underside of the cut due to three reasons: (a) the temperature gradient caused by the laser beam, (b) the beam divergence resulting in larger kerf, and (c) gas jet turbulences which allow dross to adhere. Teixidor et al. [17] geometrically characterized dross height based on energy balances in fiber laser cutting. Muhammad et al. [11] reported a qualitative study of dross deposition for dry and wet cutting conditions. From this research, it was concluded that dross deposition was higher at low cutting speed (500 mm/min) and not significant above 1000 mm/min. 

Table 1 summarizes the studies in fiber laser microcutting with stainless steel and other alloys for raw material thicknesses less than 1 mm and a laser wavelength of ~1064 nm. Although several studies deal with surface roughness and dross adherence, there is no reported research focusing on the quantification of dross adherence on the opposite wall of the miniature tube. These quality parameters are critical in the surface finish of permanently implanted devices, such as coronary stents, among others [18]. 

This research is focused on assessing the influence of cutting process parameters on surface roughness ($R_{a}$) and back wall dross ($D_{\mathrm{bw}}$). Fiber laser microcutting experimentation was conducted on 1.8 mm diameter miniature tubes (material: AISI 316L stainless steel) with two different dimensions of wall thickness ($t_{w}$) and two hardness conditions as follows: annealed with 110 μm wall thickness and hard drawn with 160 μm wall thickness.

## 2. Materials and Methods

**Figure 1:**
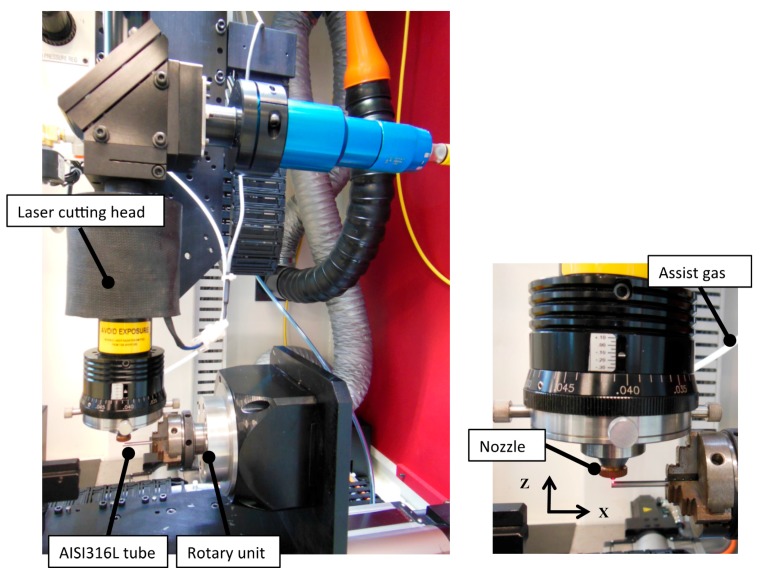
Experimental setup.

A 4-axis laser-cutting machine (PRECO Model MedPro ST2000, Preco Inc, Wisconsin, WI, USA) was used to manufacture a single strut of a coronary stent. This machine uses an IPG fiber laser model YLR-150/1500-QCW-AC (IPG Photonics, Oxford, MA, USA). Experiments were conducted with continuous wave (CW) operation in a modulated mode, using a fiber feeding of 50 μm core diameter, a 120 mm collimator and final focus lens of 50 mm, resulting in a theoretical spot size of 20.8 μm. However, according to the beam analysis provided for the machine supplier [23] the minimum radius is of the order ~32.1 μm. Table 2 indicates the fiber laser system specifications used in this research. Figure 1 illustrates the experimental setup used in this research with a close-up image of the laser cutting head. The tubes were held by the rotating chuck in close proximity to avoid deflections during the process. Experiments were performed using stainless steel tubes (AISI 316L) with an outer diameter of 1.8 mm, and a wall thicknesses of 110 and 160 µm. Table 3 presents the chemical composition of AISI 316L, while the mechanical properties (data were provided by material supplier: Minitubes, Grenoble, France) [24] and metallographies are presented in Table 4.

From images in Table 4, a small grain size was observed in the hard drawn processed tube compared to the annealed tube. Before struts were laser cut, the tubes were pre-cleaned with a solution of 10% ethanol and distilled water in ultrasonic bath and then dried. 

The experimental design was a two-level full factorial 2^5^ with four central points and three replications for each parameter combination. Separate experimental designs were applied for each tube thickness and hardness condition. An analysis of variance (ANOVA) was performed to evaluate the process parameter’s significance for each kind of miniature tube. Table 5 depicts the five experimental factors investigated, as well as their corresponding high and low levels. The experimental responses of interest were average surface roughness and back wall dross percentage.

Average surface roughness was measured over the laser cut surface on the Y-Z plane with a confocal microscope (Zeiss Model CSM 700, Carl Zeiss AG, Oberkochen, Germany) according to the ISO 4288 standard. The measurement procedure consisted of obtaining a measurement on the cut edge using a sample basic length of 0.8 mm and tracing an evaluation line of 4 mm. Back wall dross percentage was assessed by measuring a total area of 1080 µm by 780 µm in the middle of the miniature tube on the X-Y plane with a stereomicroscope (Zeiss Model Discovery V8, Carl Zeiss AG, Oberkochen, Germany). The reference area for back wall dross measurement was selected based on the miniature tube dimensions and laser trajectory in the cut geometry.

**Figure 2:**
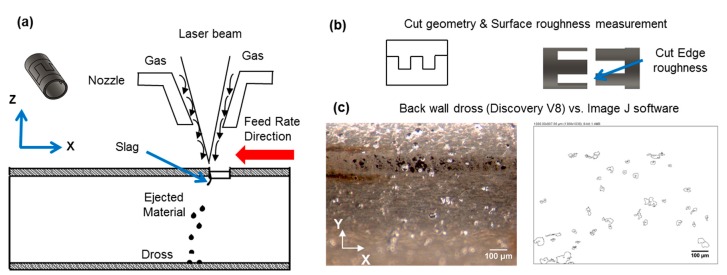
Back wall dross and surface roughness measurement. (**a**) Schematic representation of laser beam action and dross deposition on back wall. (**b**) Drawing of the cut geometry and representation of the surface roughness measurement on the cut edge. (**c**) Macrograph of the analyzed back wall area with adhered dross (Discovery V8) versus processed image (ImageJ) showing the area of individual dross particles.

ImageJ software (National Institutes of Health, Bethesda, MD, USA) was used for the image processing of the back-wall dross measurements. Back wall dross was measured as the percentage of the area covered by adhered particles over the whole measured area using the “analyze particle” command and particle diameter range of 16 µm to 100 µm. Figure 2 presents a drawing of (a) the laser beam phenomena, (b) the cut geometry which consists of two separable pieces to measure the surface roughness on the cut edge, and (c) the dross adhered at the back wall of the tube.

## 3. Results

The average surface roughness and back wall dross percentage were analyzed through an ANOVA to assess the statistical significance of laser cutting process parameters. Table 6 and Table 7 present the results of the analysis of variance in terms of coded factors. The most statistically significant factors (*p*-value < 0.05) are highlighted. The complete data set is show in the Table A1 and Table A2 in Appendix A.

Statistical models for average surface roughness and back wall dross were developed for each type of miniature tube. Equations (1) and (2) present the models for average surface roughness ($y_{1}$) (units in μm) and back wall dross ($y_{2}$) (units in %) for the miniature tube A with 110 µm wall thickness. Experimental factors *X*_1_ through *X*_5_ are physically independent and coded as −1 for low level and +1 for high level. For miniature tube A, the surface roughness ($R_{a}$) model has an R-sq (adjusted) of 85.48, while the back wall dross model has an R-sq (adjusted) of 87.9.

(1) $$y_{1}=1.071+0.046X_{1}+0.040X_{2}+0.040X_{3}+0.040X_{4}+0.043X_{5}+0.043X_{1}X_{2}+0.026X_{1}X_{4}$$

(2) $$y_{2}=4.885+0.468X_{2}+0.292X_{3}+0.9933X_{4}+0.496X_{5}+0.276X_{1}X_{2}+0.274X_{1}X_{5}$$

Furthermore, for the hard drawn miniature tube B with 160 µm wall thickness, the model for surface roughness ($R_{a}$) and back wall dross have an R-sq (adjusted) of 84.55 and 92.27, respectively. Equations (3) and (4) present the models for average surface roughness ($Y_{1}$) and back wall dross ($Y_{2}$) for miniature tube type B. These models are predictive statistical models used to evaluate surface roughness and back wall dross according to a reduced model. For example, in the miniature tube A, $X_{1}$ had the greatest effect on surface roughness while $X_{4}$ followed by $X_{5}$ resulted with the greatest effect for back wall dross response. Although, these models have a good agreement, curvature is significant due to interactions between cutting parameters and the central point, therefore further studies should require the study of models with axial points as a surface response design.

(3) $$Y_{1}=1.06+0.052X_{2}+0.066X_{4}+0.0411X_{5}+0.045X_{1}X_{4}+0.034X_{1}X_{5}+0.048X_{4}X_{5}$$

(4) $$Y_{2}=2.502+0.328X_{5}-0.307X_{4}X_{5}$$

The experimental results show a number of interactions among factors. These interactions are correlated with the multiple physical phenomena occurring during laser melting, such as the fluid dynamics of the molten material being pushed away by the assist gas pressure, thermal effects, and chemical reactions between the material and the assist gas. 

**Figure 3:**
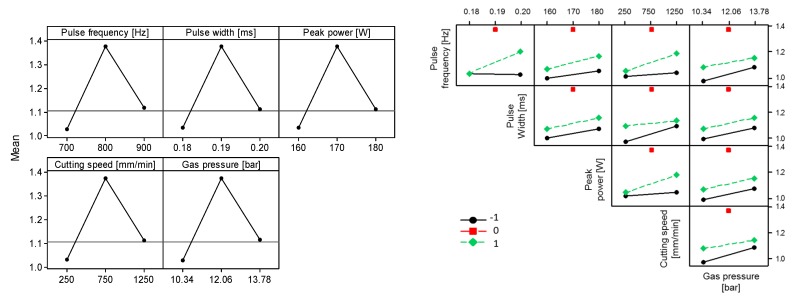
Main effects and interaction plots for surface roughness (miniature tube type A).

**Figure 4:**
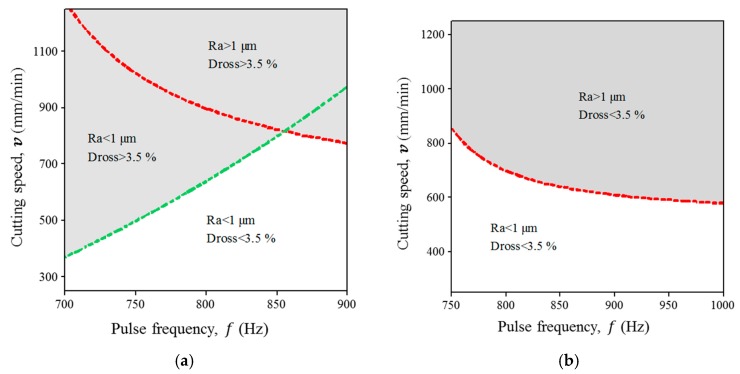
Influence of pulse frequency and cutting speed on surface roughness and back wall dross. (**a**) Miniature Tube A: Annealed and 110 µm thickness (fixed parameters: pulse width, 0.18 ms; peak power, 160 W; gas pressure, 10.34 bar). (**b**) Miniature tube B: Hard drawn and 160 µm thickness (fixed parameters: pulse width, 0.19 ms; peak power, 170 W; gas pressure, 12.06 bar).

**Figure 5:**
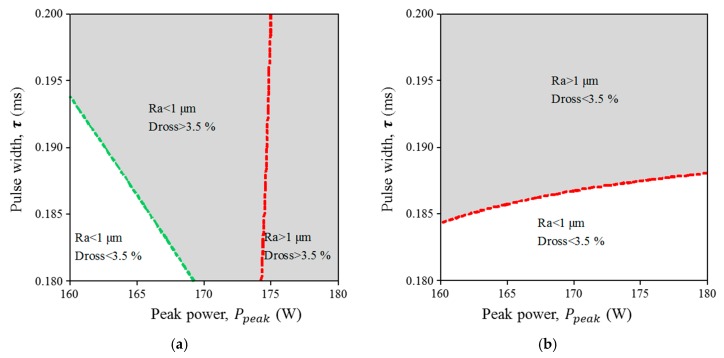
Influence of pulse width and peak power on surface roughness and back wall dross. (**a**) Miniature tube A: Annealed and 110 µm thickness (fixed parameters: pulse frequency, 700 Hz; cutting speed, 250 mm/min; gas pressure, 10.34 bar). (**b**) Miniature tube B: Hard drawn and 160 µm thickness (fixed parameters: pulse frequency, 875 Hz; cutting speed, 750 mm/min; gas pressure, 12.06 bar).

As an example, the main effects and interaction plots are shown in Figure 3 for surface roughness in miniature tube A (annealed tube). Based on this analysis, fixed parameters in overlay contour plots were selected with settings that tend to decrease surface roughness and back wall dross (see Figure 4 and Figure 5). The potential correlation between surface roughness and back wall dross was checked. Under the range of experimental conditions under study, there is no correlation between these two quality parameters.

Overlay plots were developed in order to better illustrate the relationship between process factors and responses. These plots made use of average surface roughness ($R_{a}$) and back wall dross responses ($D_{\mathrm{bw}}$). For the miniature tube type A (1.8 mm outside diameter (OD) and 110 μm wall thickness), the low levels of experimental factors were used as fixed parameters (see Table 5) in order to illustrate minimum values in both responses. In contrast, the intermediate levels of experimental factors (see Table 5) were used for the illustration of responses in the miniature tube type B (1.8 mm OD and 160 μm wall thickness). 

**Figure 6:**
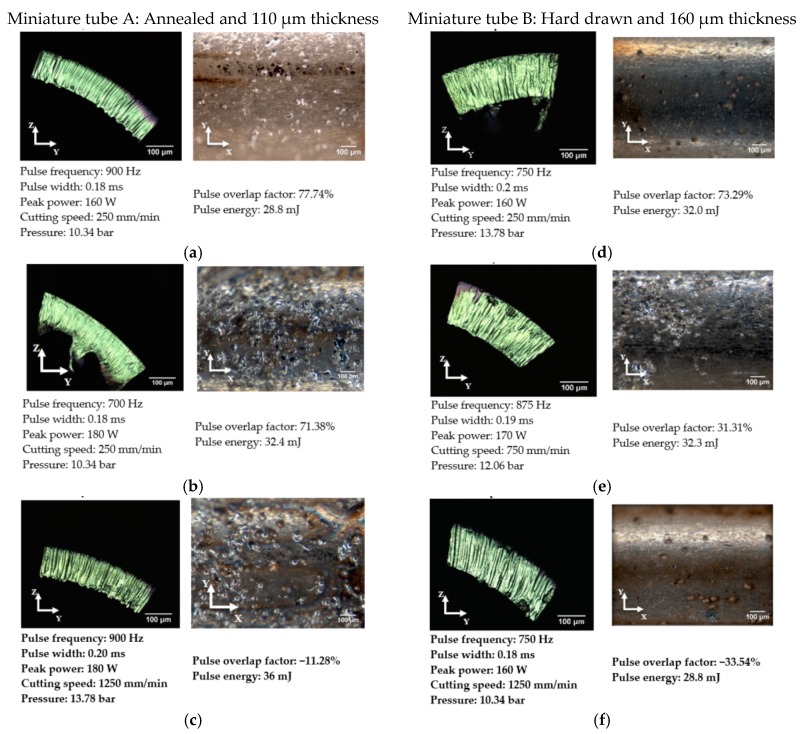
Average surface roughness and back wall dross images. (**a**) Ra < 1 μm and Back Wall Dross < 3.5%. (**b**) Ra < 1 μm and Back Wall Dross > 3.5%. (**c**) Ra > 1 μm and Back Wall Dross > 3.5%. (**d**) Ra < 1 μm and Back Wall Dross < 3.5%. (**e**) Ra < 1 μm and Back Wall Dross > 3.5%. (**f**) Ra > 1 μm and Back Wall Dross < 3.5% .

**Figure 7:**
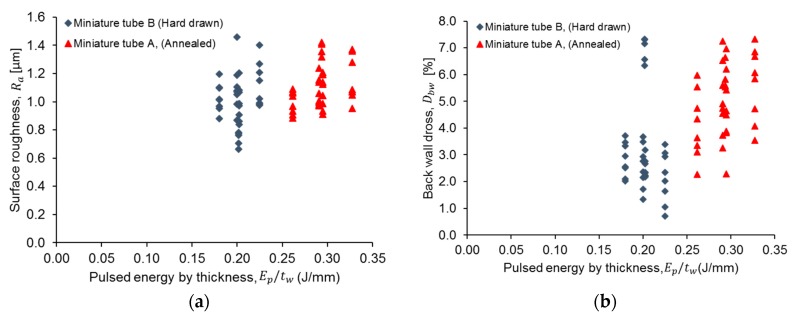
Influence of pulse energy by thickness on (**a**) Surface roughness and (**b**) back wall dross for miniature tube A and B.

Further analysis is shown in Figure 6 for selected combinations of process parameters. For a quantitative comparison between the miniature tubes type A vs. type B, the pulse laser energy was normalized for the wall thickness of the tube and plotted for surface roughness and back wall dross. Results are presented in Figure 7. (For a full set of results, see Table A1 and Table A2).

**Figure 8:**
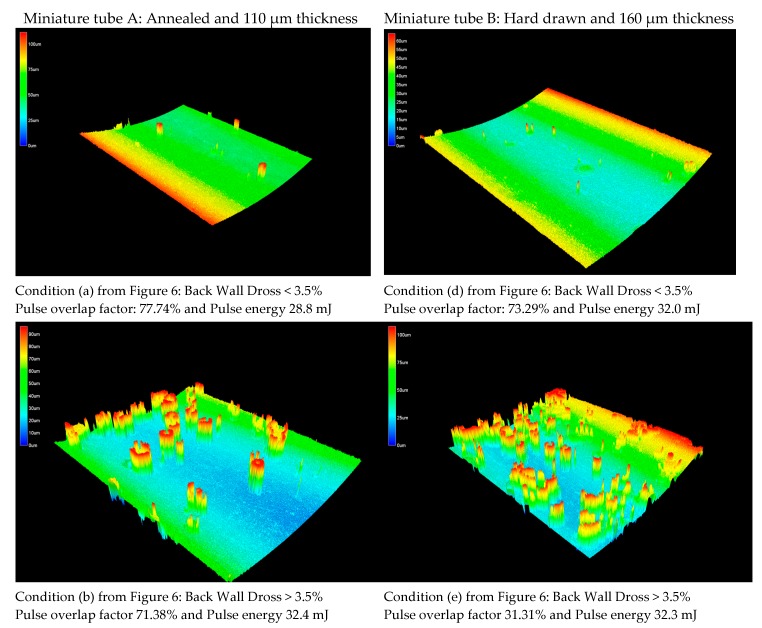
Confocal microscopy images of back wall surface topography. In condition (**a**): Back Wall Dross < 3.5%, condition (**b**): Back Wall Dross > 3.5%, condition (**d**): Back Wall Dross < 3.5%, and condition (**e**): Back Wall Dross > 3.5%.

A more detailed topography for the back wall dross is given in Figure 8 for conditions (a), (b), (d) and (e) from Figure 6. In laser processing, a better analysis of the effect of interacting parameters can be done when some parameters are combined. Pulse overlapping factor, $O_{f}$, is associated with the periodic striation on the cut edge produced in pulsed mode and given by Equation (5), where v is the cutting speed, f is the pulse frequency, and d is the theoretical spot diameter, while

(5) $$O_{f}=100\left(1-\frac{v}{fd}\right)$$

pulse energy ($E_{p}$) is given by Equation (6), where $P_{\mathrm{peak}}$ is peak power and τ pulse width [25].

(6) $$E_{p}=P_{\mathrm{peak}}\;\tau$$

The corresponding values of overlapping factor ($O_{f}$) and pulse energy ($E_{p}$) are indicated for selected combinations of process parameters in Figure 6. It is observed that back wall dross higher than 3.5% occurs only with a pulse energy value higher than 32 mJ (see conditions b, c, and e in Figure 6). In addition, the use of high pulse overlap factor and low pulse energy generates a combination of low average surface roughness and small percentage of back wall dross (see conditions (a) and (d) in Figure 6). Conditions (c) and (f) in Figure 6 show a negative overlap factor. These conditions imply a series of separated laser beam pulses, creating circular notches. Therefore, the strut was separated mechanically, causing irregularities on surface. 

## 4. Discussion

White zones in Figure 4 and Figure 5 denote the areas where surface roughness (less than 1 µm) and back wall dross (less than 3.5%) are decreased, representing the best configuration of process parameters for both responses. These zones were defined as the value below 70% of the maximum result in surface roughness and 50% of the maximum result in back wall dross for both types of tubes. Wandera et al. reported that the best quality in fiber laser cutting of stainless steel is found using the lowest level of cutting speed [26]. The results shown in Figure 6a,b are consistent with Wandera et al. Also, Mahrle et al. described the achievable cutting speed according to the inclination cutting front in the laser fusion process, which depends on the sheet thickness, absorptivity and the energy required to melt a volume per unit of time [27]. Their results indicate that a thicker sheet requires control of the cut front inclination (sheet thickness up to 2.5 mm). Further studies should clarify this phenomena in thinner wall thickness, such as those used for coronary stents. In a different study, according to Thawari et al. [28], the periodic striations on laser cutting affect cut quality attributes like surface roughness and kerf width. As the pulse overlap factor decreases, the kerf width tends to decrease and the surface roughness tends to increase. Similarly, in this study, a low value of pulse overlap factor is correlated with higher surface roughness (see condition e in Figure 5). 

Tubes used for surgical applications are manufactured by methods described in ASTM F225 [29]. The specific manufacturing methods can be classified as cold drawn and annealing processes. The materials used in this research were certified materials intended for the manufacturing of coronary stents. It is well known that the fabrication of coronary stents includes several steps such as laser cutting, electropolishing, passivation and annealing process [30]. The Food and Drug Administration (FDA) recommends the tracking of the stress history of the coronary stents, including the manufacturing (fabrication, annealing, electropolishing, heat setting, etc.), crimping, expansion/deployment, stent recoil and physiologic loading conditions [31]. 

In this work, laser cutting was studied with miniature tubes manufactured by annealing and hard drawing processes. The annealing process is an effective method for softening the stent material [32], which provides high ductility even at high strain amplitudes, compared with the hard drawing process characterized by a low ductility and high strength. Poncin et al. explained that although a fully annealed condition is desirable, a cold worked tube is preferred in order to reduce the risk of handling damage when the tube is cut. Certainly, after laser cutting and electropolishing, an annealing step is recommended to release the residual stresses during laser cutting and to control the mechanical properties and microstructure [33]. 

According to our results, as reported in Figure 7, the manufacturing process (i.e., annealing vs. hard drawn) can affect some quality related parameters. Lavvafi H. et al., described the effects of laser treatment on AISI 316L wires fabricated by the hard drawing and annealed processes [34]. Their results indicated that surface roughness on annealed tubes is directly related to the laser power level. In addition, laser processing shows an effect on the bulk mechanical properties of hard drawn wire (e.g., reduction in ultimate tensile strength (UTS) and micro hardness). From Figure 7, our surface roughness results are dependent on five process parameters. Therefore further studies should be performed in regards to the influence of the laser energy on the surface finish and mechanical performance of the stent. Also, it was observed that the amount of back wall dross tends to increase in the case of the annealed tubes. This implies that the annealed miniature tubes will require more work post processing in order to eliminate this residue. For the harder condition derived from hard drawing, there is also the risk of cracking due to temperature gradients. In this study, there was no evidence of cracking.

In particular, surface roughness and back wall dross have implications on the coronary stent quality. Muhammad et al. explored an underwater technique to drag the dross particles inside AISI 316L tubes, reducing the effects of back wall damage and cracks caused by the heat dissipation [11]. The hydrodynamics of water over the workpiece surface in laser cutting was explained as a thin and thermally disturbed water layer which prevented the redeposition of the melted material, thus avoiding the formation of debris and a recast layer [35]. In a related study, Demir et al. presented the influence of submerging AZ31 alloy in three different liquids (water, alcohol and oil) to get a dross-free cutting. An alcohol-water solution showed the best results in terms of chemical dross dissolution [36]. In the current study, the surface topography images (Figure 8) showed a reduction of the dross formations (conditions (a) and (d)) with low levels of pulse energy, avoiding the melting and thermal effects on surface. However, these surfaces were not free of dross, therefore the use of the dragging techniques could be of interest in order to reduce the molten deposits. Also, back wall damage can be related to the tube thicknesses (i.e., thicker material should require higher energy). However this comparison is not conclusive due to the differences in tube processing. 

In related studies, the potential of femtosecond laser machining has been demonstrated in the case of extremely delicate materials (e.g., polymeric stents and biodegradable stents) [37] and sensitive materials (shape memory alloys) due to the reduction in thermal effects during laser machining [38]. Biffi et al. performed a comparison between a CW, or long pulsed laser, and a femtosecond laser for Nitinol with thicknesses of 100 μm and 130 μm. From their results, the CW laser showed a large amount of material deposited in the form of drops, while the femtosecond laser samples appeared smother and the kerf was more precise and regular [39]. However, currently coronary stents from metallic alloys (e.g., stainless steel and CoCr) are industrially manufactured with lasers under longer pulse.

## 5. Conclusions and Future Work

This research is focused on assessing the influence of process parameters on average surface roughness (*R*_a_) and back wall dross during the fiber laser microcutting of miniature tubes, with potential application in medical implants such as coronary stents. Fiber laser microcutting experimentation was conducted on 1.8 mm diameter miniature tubes (material: AISI 316L stainless steel) with two different dimensions of wall thickness and hardness conditions (type A: annealed with 110 μm wall thickness and type B: hard drawn with 160 μm wall thickness). Hard drawn tubes showed better results in terms of back wall dross when compared to the miniature tubes under the annealed condition. 

A quantitative evaluation of the back-wall dross adherence phenomenon was carried out which, to our best knowledge is the first of its kind. Through the appropriate combination of process parameters (i.e., high level of pulse overlapping factor and pulse energy below 32 mJ) it is possible to achieve less than 1 μm in Ra at the edge of the laser-cut tube and less than 3.5% dross deposits at the back wall of the miniature tube. 

In addition, a high degree of interaction is present among the studied process parameters (i.e., pulse frequency, pulse width, peak power, cutting speed and gas pressure), together with the central point curvature. Therefore, further analysis is required in order to develop a more robust statistical model which would reduce the number of process variables and exploring spot overlap and pulse energy parameters to appropriately predict quality indicators in the fiber laser micro-cutting of miniature tubes.

## Figures and Tables

**Table 1:** State-of-the-art of fiber laser microcutting with stainless steel and other alloys.

Reference	Alloy	Raw Material Shape (Thickness)	Cut Geometry	Surface Topography Response
Liu, L. et al., 2017 [19]	Cobalt-chromium (CoCr)	Tube, outside diameter (OD) = 2.0 mm (150 µm)	Ring with grooves	Kerf width and Surface topography
Teixidor et al., 2014 [17]	AISI 316L stainless steel	Sheet (100 µm)	Stent mesh	Surface roughness and edge dross
Demir A.G et al., 2014 [20]	AZ31 magnesium	Tube, OD = 2.5 mm (200 µm) Sheet (400 µm)	Stent mesh	Kerf width, taper angle, Surface roughness
Demir A.G et al., 2013 [21]	AZ31 magnesium	Tube, OD = 2.5 mm (200 µm)	Stent mesh	Surface roughness with kerf quality
Biffi C.A. et al., 2014 [22]	NiTiCu alloy	Sheet (150 µm)	Linear cuts	HAZ, hardness, chemical composition
Adelmann et al., 2011 [14]	Aluminum	Sheet (1 mm)	Kerf width	Burr height
Muhammad et al., 2010 [11]	AISI 316L stainless steel	Tube, OD = 3.175 mm (150 µm)	Stent-Strut	Surface roughness with back wall dross
Meng et al., 2009 [2]	AISI 316L stainless steel	Tube, OD = 2 mm (110 µm)	Kerf width	n/a
Sobih et al., 2008 [6]	EN43 annealed mild steel	Sheet (1 mm)	Kerf width	Surface roughness with striations
Baumeister et al., 2006 [8]	1.4301 stainless steel, AISI 304 equivalent	Sheet (100, 200 and 300 µm)	Kerf width	n/a
Kleine et al., 2002 [10]	AISI 316L Stainless steel AISI 316L	Sheet (100 µm approx.)	n/a	Surface roughness and Recast

**Table 2:** Fiber laser system specifications.

Parameter	Specification	Unit
Nozzle diameter	0.50	mm
Standoff distance	0.25	mm
Operation mode	CW/pulsed	-
Maximum peak power	1500	W
Maximum average power (CW mode)	250	W
Minimum pulse width (CW mode and modulated)	0.010	ms
Maximum average power (pulsed mode)	150	W
Pulse width (pulsed mode)	0.2–10	ms
Wavelength (λ)	1070	nm
Beam parameter product	0.96	mm mrad
Beam quality, M^2^	2.82	-

**Table 3:** Chemical composition AISI 316L for two kinds of miniature tube with 1.8 mm in diameter [24].

Tube Type	C	Si	P	S	Mn	Ni	Cr	Mo	Fe	Cu	N
Miniature tube A *	0.011	0.41	0.014	<0.002	1.93	14.73	17.61	2.71	Bal.	0.08	0.04
Miniature tube B **	0.019	0.42	0.016	<0.002	1.87	14.84	17.53	2.72	Bal.	0.08	0.04

* Miniature tube A: Annealed condition and 110 μm wall thickness; ** Miniature tube B: Hard drawn condition and 160 μm wall thickness

**Table 4:** Mechanical properties of miniature tube materials [24].

Mechanical Properties	Miniature Tube A *	Miniature Tube B **
Metallography	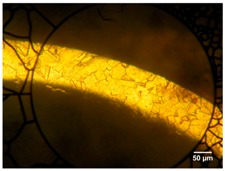	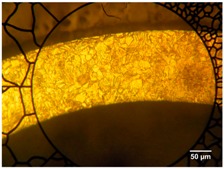
Grain size	7	7–8
Hardness, HDN (HV0.3)	190.0	345.0
Yield strength 0.2%, $\sigma_{Y}$ (MPa)	315.9	753.5
Ultimate tensile strength, $\sigma_{\mathrm{UTS}}$ (MPa)	600.2	943.7
Young’s modulus, *E* (GPa)	200.0	240.0

* Miniature Tube A: Annealed 1.8 mm OD and 110 µm wall thickness; ** Miniature Tube B: Hard Drawn 1.8 mm OD and 160 µm wall thickness.

**Table 5:** Experimental factors for DOE.

Factors		Miniature Tube A *		Miniature Tube B **	
		Low Level	High Level	Low Level	High Level
DOE Code		−1	+1	−1	+1
$X_{1}$	Pulse frequency, f (Hz)	700	900	750	1000
$X_{2}$	Pulse width, τ (ms)	0.18	0.20	0.18	0.20
$X_{3}$	Peak power, $P_{\mathrm{peak}}$ (W)	160	180	160	180
$X_{4}$	Cutting speed, v (mm/min)	250	1250	250	1250
$X_{5}$	Assist Gas $N_{2}$ Pressure, P (Bar)	10.34	13.78	10.34	13.78

* Miniature Tube A: Annealed 1.8 mm OD and 110 µm wall thickness; ** Miniature Tube B: Hard Drawn 1.8 mm OD and 160 µm wall thickness.

**Table 6:** Analysis of variance (ANOVA)—Miniature tube A *.

Source	Average Surface Roughness (*R*_a_)				Back Wall Dross			
	DF	SS	F	P	DF	SS	F	P
*X*_1_: Pulse fequency, *f* (Hz)	1	0.06833	13.72	**0.001**	1	0.3336	1	0.328
*X*_2_: Pulse width, *τ* (ms)	1	0.05179	10.4	**0.004**	1	7.0369	21.01	**0.000**
*X*_3_: Peak power, *P*_peak_ (W)	1	0.05132	10.31	**0.004**	1	2.7396	8.18	**0.009**
*X*_4_: Cutting speed, *v* (mm/min)	1	0.05239	10.52	**0.003**	1	31.5700	94.25	**0.000**
*X*_5_: Pressure, *P* (bar)	1	0.06097	12.25	**0.002**	1	7.8900	23.57	**0.000**
*X*_1_*X*_2_	1	0.60109	12.07	**0.002**	1	2.4436	7.3	**0.012**
*X*_1_*X*_4_	1	0.02198	4.41	**0.046**	1	0.4157	1.24	0.276
*X*_1_*X*_5_	1	0.00237	0.48	0.497	1	2.4123	7.2	**0.013**
*X*_2_*X*_3_	1	0.00043	0.09	0.772	1	0.0016	0	0.946
*X*_4_*X*_5_	1	0.00388	0.78	0.386	1	0.0944	0.28	0.600
Curvature	1	0.32965	66.2	**0.000**	1	3.6300	10.84	**0.003**
Residual error	24	0.11950	-	-	24	8.0389	-	-
Lack of fit	21	0.11254	2.31	0.268	21	7.2449	1.3	0.475
Pure error	3	0.00696	-	-	3	0.7940	-	-

* Miniature Tube A: Annealed 1.8 mm OD and 110 µm wall thickness (*p*-values below 0.05 are bolded).

**Table 7:** ANOVA—Miniature tube B *.

Source	Average Surface Roughness (*R*_a_)				Back Wall Dross			
	DF	SS	F	P	DF	SS	F	P
*X*_1_: Pulse Fequency, *f* (Hz)	1	0.01477	2.14	0.156	1	0.022	0.07	0.794
*X*_2_: Pulse width, *τ* (ms)	1	0.086544	12.52	**0.002**	1	0.76	2.39	0.135
*X*_3_: Peak Power, *P*_peak_ (W)	1	0.00044	0.06	0.803	1	1.146	3.6	0.069
*X*_4_: Cutting Speed, *v* (mm/min)	1	0.14007	20.26	**0.000**	1	0.401	1.26	0.272
*X*_5_: Pressure, *P* (bar)	1	0.05401	7.81	**0.010**	1	3.457	10.87	**0.003**
*X*_1_*X*_2_	1	0.01165	1.68	0.206	1	0.662	2.08	0.161
*X*_1_*X*_4_	1	0.0651	9.42	**0.005**	1	0.206	0.65	0.428
*X*_1_*X*_5_	1	0.03812	5.52	**0.027**	1	0.052	0.16	0.689
*X*_2_*X*_3_	1	0.00794	1.15	0.294	1	0.007	0.02	0.881
*X*_4_*X*_5_	1	0.07538	10.9	**0.003**	1	3.0283	9.53	**0.005**
Curvature	1	0.4514	65.34	**0.000**	1	85.186	267.97	**0.000**
Residual error	25	0.17281			25	7.9474		
Lack of fit	21	0.162285	2.94	0.153	21	7.107	1.61	0.347
Pure error	4	0.010528			4	0.841		

* Miniature tube B: Hard drawn 1.8 mm OD and 160 µm wall thickness (*p*-values below 0.05 are bolded).

## Appendix A

**Table A1:** Complete data set for miniature tube type A *.

*X*_1_	*X*_2_	*X*_3_	*X*_4_	*X*_5_	Average Surface Roughness $R_{a}$ (mm)	Back Wall Dross responses $D_{bw}$ (%)
−1	−1	−1	−1	−1	0.91	3.10
1	−1	−1	−1	−1	0.97	2.26
−1	1	−1	−1	−1	0.97	3.25
1	1	−1	−1	−1	0.99	3.73
−1	−1	1	−1	−1	0.91	3.84
1	−1	1	−1	−1	0.93	2.28
−1	1	1	−1	−1	1.05	4.72
1	1	1	−1	−1	1.07	3.55
−1	−1	−1	1	−1	0.88	5.53
1	−1	−1	1	−1	1.04	4.34
−1	1	−1	1	−1	0.98	4.92
1	1	−1	1	−1	1.15	5.60
−1	−1	1	1	−1	1.13	5.43
1	−1	1	1	−1	1.14	4.49
−1	1	1	1	−1	0.95	7.33
1	1	1	1	−1	1.36	5.85
−1	−1	−1	−1	1	1.07	3.63
1	−1	−1	−1	1	0.93	3.35
−1	1	−1	−1	1	1.06	4.55
1	1	−1	−1	1	1.24	4.73
−1	−1	1	−1	1	1.04	3.88
1	−1	1	−1	1	0.99	4.65
−1	1	1	−1	1	1.07	4.07
1	1	1	−1	1	1.28	6.69
−1	−1	−1	1	1	1.09	5.98
1	−1	−1	1	1	1.06	4.74
−1	1	−1	1	1	1.00	6.53
1	1	−1	1	1	1.14	7.24
−1	−1	1	1	1	1.19	6.97
1	−1	1	1	1	1.21	6.20
−1	1	1	1	1	1.09	6.07
1	1	1	1	1	1.37	6.85
0	0	0	0	0	1.35	5.82
0	0	0	0	0	1.32	5.53
0	0	0	0	0	1.42	6.65
0	0	0	0	0	1.41	5.59

***** Miniature tube type A: X_1_: Pulse Frequency *f* (Hz); X_2_: Pulse Width τ (ms); X_3_: Peak Power *P*_peak_ (W); X_4_: Cutting Speed *v* (mm/min); X_5_: Pressure *P* (bar).

**Table A2:** Complete data set for miniature tube type B.

*X*_1_: Pulse Frequency *f* (Hz)	*X*_2_: Pulse Width τ (ms)	*X*_3_: Peak Power *P*_peak_ (W)	*X*_4_: Cutting Speed *v* (mm/min)	*X*_5_: Pressure *P* (bar)	Average Surface Roughness $R_{a}$ (mm)	Back Wall Dross Responses $D_{bw}$ (%)
−1	−1	−1	−1	−1	1.02	2.02
1	−1	−1	−1	−1	0.97	2.09
−1	1	−1	−1	−1	1.05	1.72
1	1	−1	−1	−1	0.98	1.34
−1	−1	1	−1	−1	0.97	2.35
1	−1	1	−1	−1	0.84	2.16
−1	1	1	−1	−1	1.21	1.64
1	1	1	−1	−1	0.98	0.71
−1	−1	−1	1	−1	1.10	2.55
1	−1	−1	1	−1	0.88	2.52
−1	1	−1	1	−1	1.08	2.76
1	1	−1	1	−1	1.19	3.49
−1	−1	1	1	−1	0.86	2.76
1	−1	1	1	−1	1.07	2.23
−1	1	1	1	−1	0.98	1.06
1	1	1	1	−1	1.15	3.38
−1	−1	−1	−1	1	1.10	3.71
1	−1	−1	−1	1	0.95	3.33
−1	1	−1	−1	1	0.87	2.94
1	1	−1	−1	1	1.07	3.67
−1	−1	1	−1	1	0.91	3.17
1	−1	1	−1	1	0.99	2.32
−1	1	1	−1	1	1.02	2.01
1	1	1	−1	1	0.99	3.07
−1	−1	−1	1	1	1.01	2.96
1	−1	−1	1	1	1.20	3.47
−1	1	−1	1	1	1.11	2.37
1	1	−1	1	1	1.46	2.15
−1	−1	1	1	1	1.08	2.67
1	−1	1	1	1	1.20	2.19
−1	1	1	1	1	1.27	2.93
1	1	1	1	1	1.40	2.35
0	0	0	0	0	0.77	7.16
0	0	0	0	0	0.76	6.56
0	0	0	0	0	0.66	7.33
0	0	0	0	0	0.78	7.31
0	0	0	0	0	0.71	6.34
